# Elucidating the reaction mechanism of SO_2_ with Cu-CHA catalysts for NH_3_-SCR by X-ray absorption spectroscopy†

**DOI:** 10.1039/d3sc03924b

**Published:** 2023-10-10

**Authors:** Anastasia Yu. Molokova, Reza K. Abasabadi, Elisa Borfecchia, Olivier Mathon, Silvia Bordiga, Fei Wen, Gloria Berlier, Ton V. W. Janssens, Kirill A. Lomachenko

**Affiliations:** a European Synchrotron Radiation Facility 71 avenue des Martyrs CS 40220 38043 Grenoble Cedex 9 France lomachenko@esrf.fr; b Department of Chemistry and NIS Centre, University of Turin via Giuria 7 10125 Turin Italy; c Umicore Denmark ApS Kogle Allé 1 2970 Hørsholm Denmark TonV.W.Janssens@eu.umicore.com; d Umicore AG & Co Rodenbacher Chaussee 4 63457 Hanau Germany

## Abstract

The application of Cu-CHA catalysts for the selective catalytic reduction of NO_*x*_ by ammonia (NH_3_-SCR) in exhaust systems of diesel vehicles requires the use of fuel with low sulfur content, because the Cu-CHA catalysts are poisoned by higher concentrations of SO_2_. Understanding the mechanism of the interaction between the Cu-CHA catalyst and SO_2_ is crucial for elucidating the SO_2_ poisoning and development of efficient catalysts for SCR reactions. Earlier we have shown that SO_2_ reacts with the [Cu_2_^II^(NH_3_)_4_O_2_]^2+^ complex that is formed in the pores of Cu-CHA upon activation of O_2_ in the NH_3_-SCR cycle. In order to determine the products of this reaction, we use X-ray absorption spectroscopy (XAS) at the Cu K-edge and S K-edge, and X-ray emission spectroscopy (XES) for Cu-CHA catalysts with 0.8 wt% Cu and 3.2 wt% Cu loadings. We find that the mechanism for SO_2_ uptake is similar for catalysts with low and high Cu content. We show that the SO_2_ uptake proceeds *via* an oxidation of SO_2_ by the [Cu_2_^II^(NH_3_)_4_O_2_]^2+^ complex, resulting in the formation of different Cu^I^ species, which do not react with SO_2_, and a sulfated Cu^II^ complex that is accumulated in the pores of the zeolite. The increase of the SO_2_ uptake upon addition of oxygen to the SO_2_-containing feed, evidenced by X-ray adsorbate quantification (XAQ) and temperature-programmed desorption of SO_2_, is explained by the re-oxidation of the Cu^I^ species into the [Cu_2_^II^(NH_3_)_4_O_2_]^2+^ complexes, which makes them available for reaction with SO_2_.

## Abstract 2

*In situ* XAS reveals the mechanism of SO_2_ interaction with Cu-CHA SCR catalysts.

## Graphical abstract

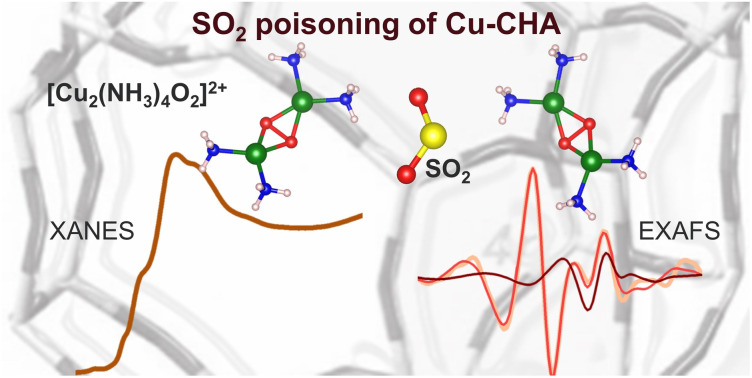

## Introduction

The current technology to reduce the harmful NO_*x*_ emissions from diesel-powered vehicles is based on the selective catalytic reduction of nitrogen oxides (NO_*x*_) to N_2_ and H_2_O by ammonia (NH_3_-SCR).^1,2^ Cu-exchanged chabazite zeolites (Cu-CHA) are preferred catalysts in diesel exhaust systems, due to their high activity in the low-temperature region (150–350 °C) and hydrothermal stability above 500 °C. The low-temperature activity of Cu-CHA-based catalysts, however, is strongly reduced in the presence of SO_2_, and therefore, application of such catalysts in exhaust systems requires the use of ultra-low sulfur diesel fuel.^3,4^

The mechanism of the NH_3_-SCR reaction in Cu-CHA based catalysts is a redox cycle,^5,6^ in which the oxidation state of Cu changes between Cu^I^ and Cu^II^. The reaction proceeds *via* a number of Cu-complexes formed by adsorption and reaction of NO, NH_3_ and O_2_ as ligands on the Cu-ions inside the CHA zeolite.^7^ The oxidation from Cu^I^ to Cu^II^ occurs by activation of O_2_, which is a crucial step in the reaction cycle. At temperatures below 250 °C, the Cu^I^ species are predominantly represented by [Cu^I^(NH_3_)_2_]^+^ complexes, which are loosely bound to the zeolite, and are therefore mobile under the reaction conditions for NH_3_-SCR.^8–10^ The O_2_ activation then takes place *via* a reaction of O_2_ with a pair of [Cu^I^(NH_3_)_2_]^+^ complexes, to form a [Cu_2_^II^(NH_3_)_4_O_2_]^2+^ complex.^6,7,11–15^ These [Cu_2_^II^(NH_3_)_4_O_2_]^2+^ complexes are then reduced back to the original [Cu^I^(NH_3_)_2_]^+^ complexes by NH_3_ and NO, under the formation of the reaction products N_2_ and H_2_O. The mobility of the [Cu^I^(NH_3_)_2_]^+^ complexes is important, as it facilitates the formation of the required Cu^I^ pairs for the O_2_ activation, enabling the NH_3_-SCR reaction at low temperatures.

Because the presence of SO_2_ results in a significantly lower activity of the Cu-CHA catalysts below 300 °C, the SO_2_ must affect the NH_3_-SCR reaction cycle. We have recently shown that SO_2_ reacts with [Cu_2_^II^(NH_3_)_4_O_2_]^2+^ complexes that are formed upon activation of O_2_.^16^ That reaction results in the decomposition of the [Cu_2_^II^(NH_3_)_4_O_2_]^2+^ complex, and a partial reduction of Cu^II^ to Cu^I^. To determine the effect of this reaction of SO_2_ with the [Cu_2_^II^(NH_3_)_4_O_2_]^2+^ complex on the rate of the NH_3_-SCR reaction, we need to know how this reaction proceeds and what reaction products are formed. A recent theoretical study proposes that deactivation by SO_2_ occurs *via* the accumulation of ammonium bisulfate (NH_4_)HSO_4_ in the zeolite after initial reaction with the [Cu_2_^II^(NH_3_)_4_O_2_]^2+^ complexes, thus limiting the mobility of the [Cu^I^(NH_3_)_2_]^+^ species, and the Cu-pair formation necessary for O_2_ activation.^17^ Such a mechanism is consistent with the observations that some SO_2_ desorption occurs around 400 °C, where ammonium bisulfate decomposes, and that most of the low-temperature activity can be recovered by heating to 550 °C.^3^ Ammonium bisulfate was also suggested as one of the species forming in the catalyst cages.^18–20^

Even though the formation of ammonium bisulfate can explain certain aspects of the deactivation by SO_2_, other observations point towards the formation of species that contain both S and Cu. Indeed, the uptake of SO_2_ is often saturated at S/Cu ratios below 1,^4,19^ which suggests that the uptake of SO_2_ is limited by the amount of Cu in the catalyst. This implies a direct interaction between SO_2_ and Cu, such that a further reaction with SO_2_ is not possible. Furthermore, the release of SO_2_ from a Cu-CHA catalyst exposed to SO_2_ occurs at a slightly higher temperature as compared to a Cu-CHA catalyst with ammonium bisulfate deposited on it *via* impregnation.^16^ This indicates that a sulfate- or sulfite-like compound is formed, that is more stable than ammonium bisulfate, which would be consistent with a direct interaction of SO_2_ with Cu. Indeed, direct interaction of SO_2_ with Cu was reported to result in the formation of Cu-sulfate^19,20^ and Cu-bisulfate,^18^ or similar species. Finally, Cu-CHA catalysts typically show 10–20 times lower low-temperature activity for NH_3_-SCR when saturated with SO_2_,^3,4,20,21^ but never a complete deactivation. If deactivation were caused by accumulation of ammonium sulfate, it would be expected to be complete upon saturation with SO_2_, at least for temperatures up to the onset of the decomposition of ammonium bisulfate.

Since the first step of the SO_2_ uptake is a reaction with the [Cu_2_^II^(NH_3_)_4_O_2_]^2+^ complexes, the uptake of SO_2_ may be affected by the Cu content in the catalyst and the gas atmosphere. The propensity toward the formation of the [Cu^II^_2_(NH_3_)_4_O_2_]^2+^ complex is determined by the Cu content and partial pressure of oxygen.^4,13,22^ Furthermore, ammonia is required as well, in order to form the mobile [Cu^I^(NH_3_)_2_]^+^ and the [Cu^II^_2_(NH_3_)_4_O_2_]^2+^ complexes. The effect of ammonia is clearly demonstrated by the observation that the Cu^II^ species formed upon exposure of Cu-CHA to O_2_ at 500 °C is much less reactive towards SO_2_ than the same species exposed to NH_3_.^16^ Therefore, the impact of SO_2_ on the activity will depend on the reaction conditions.^4,13,22^

In this work, we use *in situ* X-ray absorption spectroscopy (XAS) to monitor the reaction of SO_2_ with the [Cu_2_^II^(NH_3_)_4_O_2_]^2+^ complexes in Cu-CHA catalysts with an Si/Al ratio of 6.7, and Cu contents of 3.2 wt% and 0.8 wt%. To maximize the amount of the [Cu_2_^II^(NH_3_)_4_O_2_]^2+^ complexes in the catalysts prior to the reaction with SO_2_, we expose the catalyst to O_2_ at 200 °C for 60 minutes after reduction in a mixture of NH_3_ and NO at the same temperature. This procedure leads to an almost complete conversion of Cu to [Cu_2_^II^(NH_3_)_4_O_2_]^2+^ complexes.^11,14,16^ A Cu content of 3.2 wt% is typical for technical Cu-CHA based NH_3_-SCR catalysts. At 0.8 wt%, the low-temperature activity is significantly lower, and therefore this sample represents a situation in which the formation of the [Cu^II^_2_(NH_3_)_4_O_2_]^2+^ complex becomes less efficient. The XAS at the Cu K-edge is used to obtain information on the Cu-species formed upon exposure to SO_2_. Since the reaction of the [Cu_2_^II^(NH_3_)_4_O_2_]^2+^ complex with SO_2_ leads to a partial reduction of the Cu^II^, the presence of oxygen has an effect on the resulting species after the interaction of the [Cu_2_^II^(NH_3_)_4_O_2_]^2+^ complex with SO_2_. To determine the effect of oxygen on the interaction with SO_2_, we compare the changes in the Cu K-edge XAS spectra of the [Cu_2_^II^(NH_3_)_4_O_2_]^2+^ complex during the reaction with SO_2_ alone and with an SO_2_ + O_2_ mixture. The difference in SO_2_ uptake in the presence and absence of oxygen is determined by X-ray adsorbate quantification (XAQ)^23^ and temperature programmed desorption of SO_2_ (SO_2_-TPD). These results are combined with *in situ* S K-edge X-ray absorption spectroscopy (XAS) and S Kα X-ray emission spectroscopy (XES), which provide further information about the oxidation state and local environment of the sulfur atoms during and after reaction with the [Cu_2_^II^(NH_3_)_4_O_2_]^2+^ complex.

## Experimental

The Cu-CHA catalysts were prepared by impregnation of the parent H-CHA zeolite material (Si/Al = 6.7) with the appropriate amount of an aqueous solution of Cu-nitrate. After impregnation, the samples were dried for 2 h at 90 °C, followed by 1 h of calcination at 500 °C in air to decompose the nitrates. In this study, two Cu-CHA catalysts were used: with a Cu loading of 0.8 wt% (referred to as the low-Cu sample) and with a Cu loading of 3.2 wt% (referred to as the high-Cu sample).

The general procedure to investigate how the [Cu_2_^II^(NH_3_)_4_O_2_]^2+^ complex reacts with SO_2_ is as follows. First, the [Cu_2_^II^(NH_3_)_4_O_2_]^2+^ complex is prepared by heating a fresh catalyst sample to 500 °C in 10% O_2_, followed by cooling to 200 °C, and a reduction in 500 ppm NO + 600 ppm NH_3_ at 200 °C. The [Cu^II^_2_(NH_3_)_4_O_2_]^2+^ complex is then formed by a reoxidation of the reduced catalyst in 10% O_2_ at 200 °C. After the [Cu^II^_2_(NH_3_)_4_O_2_]^2+^ complexes have been formed, the sample is exposed to either 400 ppm SO_2_ for 3 hours, or to a mixture of 360 ppm SO_2_ and 10% O_2_ at 200 °C, while monitoring the changes in the XAS spectra. All the experiments were performed under flow conditions with He as carrier gas. Total flow was 100 ml min^−1^ (for stages without SO_2_) or 50 ml min^−1^ (for stages with SO_2_).

The whole procedure was followed by *in situ* Cu K-edge XAS at the BM23 beamline of the European Synchrotron Radiation Facility (ESRF, Grenoble, France).^24^ Exposure of the [Cu_2_^II^(NH_3_)_4_O_2_]^2+^ dimer to 400 ppm SO_2_ was also monitored by *in situ* S K-edge HERFD XANES spectroscopy during a separate experiment at the ID26 beamline of the ESRF.^25^ S K-alpha XES spectra were also recorded at ID26 for the stationary points of the treatment protocol.

The evolution of S content in the catalyst during the reaction with SO_2_ was evaluated by *in situ* XAQ^23^ measurements. Total SO_2_ uptake was also independently determined by temperature programmed desorption of SO_2_ (SO_2_-TPD).

Experimental procedures are reported in more details in the ESI.†

## Results

### Exposure to SO_2_ without O_2_

To determine the effect of Cu content on the reaction of SO_2_ with the Cu-CHA, we compared the Cu K-edge spectra for the low-Cu catalyst after exposure to SO_2_ using the same pre-treatment protocols to form specific different Cu^I^ and Cu^II^ species earlier reported for the high-Cu catalyst.^16^ We observed the same general trend, which means that the [Cu_2_^II^(NH_3_)_4_O_2_]^2+^ complex is the most sensitive to SO_2_ for the low-Cu catalyst as well. The detailed results of these measurements are reported in Fig. S4 in the ESI.†

**Fig. 1:**
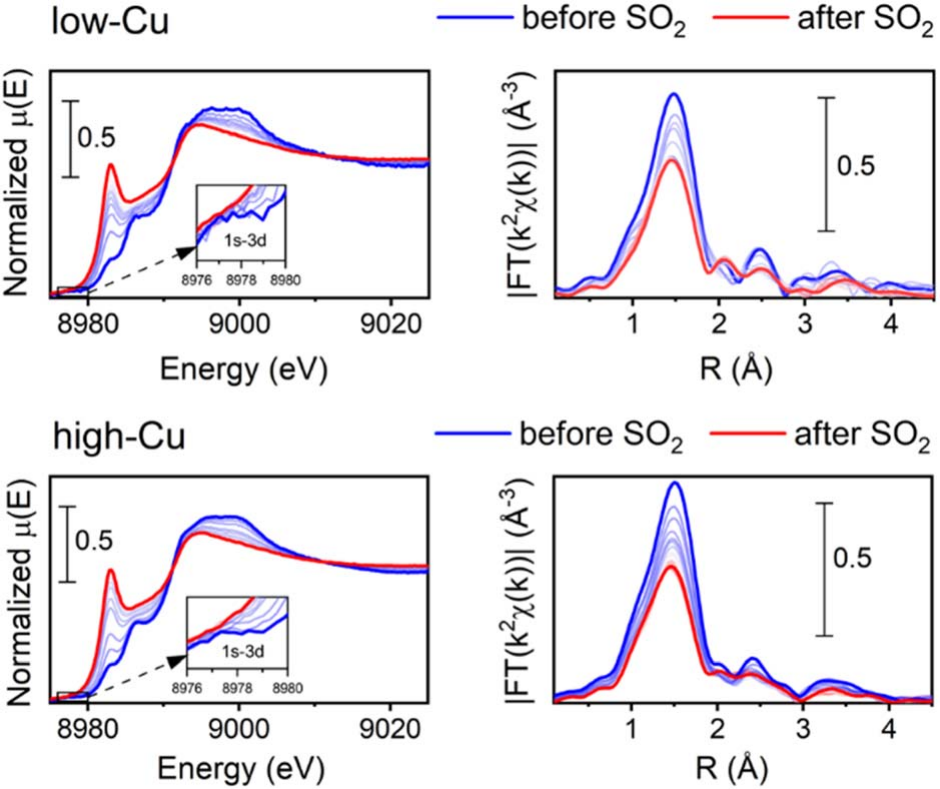
Cu K-edge XANES (a) and FT-EXAFS spectra (b) collected *in situ* during the exposure of the [Cu_2_^II^(NH_3_)_4_O_2_]^2+^ species to 400 ppm SO_2_ at 200 °C for low-Cu (0.8 wt% Cu/CHA) and high-Cu (3.2 wt% Cu/CHA) catalysts.

Fig. 1 shows the Cu K-edge XANES and EXAFS FT data collected during the exposure of the [Cu_2_^II^(NH_3_)_4_O_2_]^2+^ complex to SO_2_ at 200 °C for the low-Cu/CHA and high-Cu/CHA catalysts. The observed trends in the XANES and EXAFS spectra in these measurements are quite similar, indicating that the reaction of SO_2_ proceeds in a similar way for both catalysts. There is a clear increase of the XANES peak at 8983 eV, indicating the partial reduction of Cu^II^ to Cu^I^, and a decrease in the intensity of the first shell in the EXAFS FT, indicating a reduction of the coordination number, due to decomposition of the [Cu_2_^II^(NH_3_)_4_O_2_]^2+^ complex.

To develop a better understanding of the reaction of SO_2_ with the [Cu_2_^II^(NH_3_)_4_O_2_]^2+^ complex, we identify the reaction intermediates and reaction products by applying a combination of multivariate curve resolution alternating least squares method (MCR-ALS)^26,27^ and linear combination fitting (LCF). This hybrid approach consists of three stages. First, we apply MCR-ALS to the ensemble of the experimental spectra to deduce the shape of principal components. Then, the MCR components that can be readily associated with the known Cu species whose experimental spectra are available are substituted by the experimental Cu K-edge spectra of these species. Finally, LCF is performed over the same experimental dataset, using the experimental spectra selected at the previous step and the remaining MCR components. It allows at the same time to minimize the spectral artefacts induced by the MCR algorithm, and get a reasonable estimate of the spectra for the species that cannot be readily identified.

In the reported procedures, it was possible to select as references the experimental spectra for framework-bound Cu^II^ (fw-Cu^II^), for the [Cu^I^(NH_3_)_2_]^+^ complex, and for the [Cu_2_^II^(NH_3_)_4_O_2_]^2+^ complex. Conversely, Cu^I^ directly bound to the zeolite framework (fw-Cu^I^) was represented by calculated MCR component. The second MCR component that was used in the LCF was designated “sulfated component”, since it appeared only after the samples were exposed to SO_2_-containing mixtures.

**Fig. 2:**
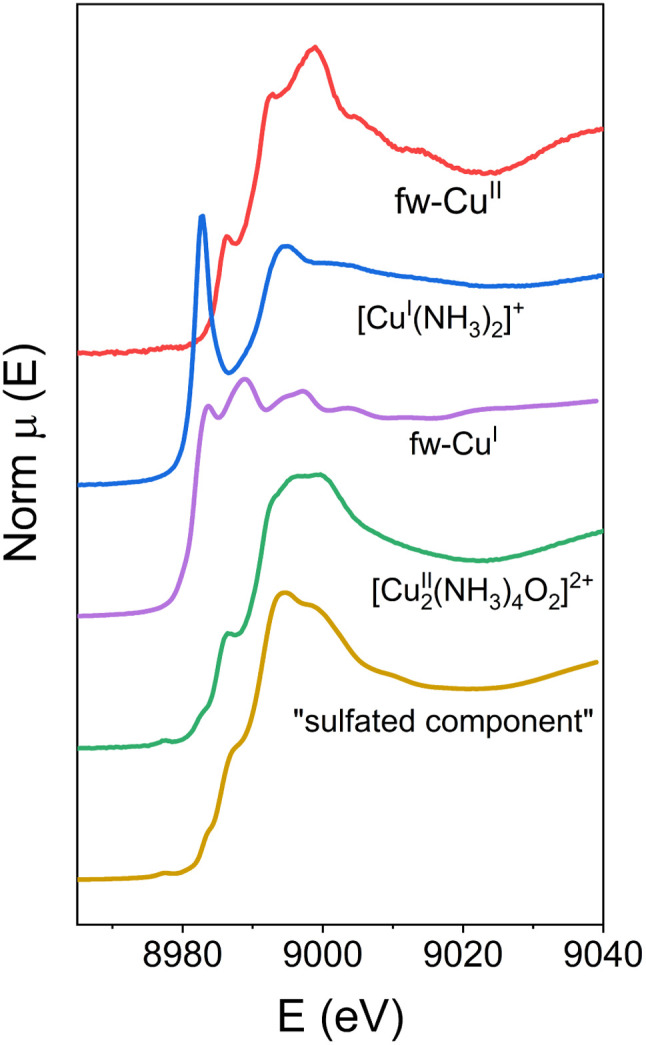
Components used as references in the linear combination fit. The fw-Cu^II^, [Cu^I^(NH_3_)_2_]^+^ and [Cu_2_^II^(NH_3_)_4_O_2_]^2+^ components are experimental spectra. The spectra of fw-Cu^I^ and the sulfated component are calculated by MCR-ALS.

Fig. 2 shows all the reference components used in the linear combination fits (three experimental spectra and two MCR components). More details on the choice of the reference spectra for the linear combination fitting procedure are given in the ESI.†

**Fig. 3:**
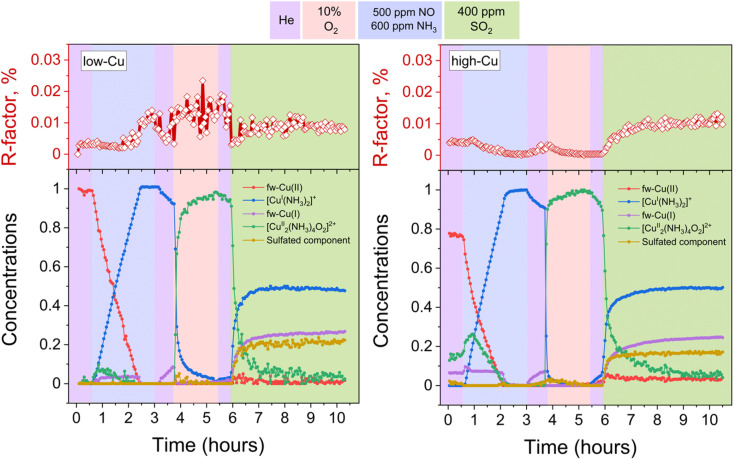
Quantification of Cu speciation during the *in situ* Cu K-edge XANES measurements from LCF, using the reference spectra shown in Fig. 2. Upper panels: *R*-factors of the linear combination fits. Lower panels: concentration profiles for the different Cu species during reduction in NH_3_ + NO, the formation of the [Cu_2_^II^(NH_3_)_4_O_2_]^2+^-complex, and exposure of the [Cu_2_^II^(NH_3_)_4_O_2_]^2+^-complex to SO_2_, for the low-Cu (left) and high-Cu (right) catalysts.

Fig. 3 presents the concentration profiles of the reference components for both the low-Cu and the high-Cu catalysts during the pre-treatment and exposure to SO_2_. The concentration of the sulfated component increases when the [Cu_2_^II^(NH_3_)_4_O_2_]^2+^-complex is exposed to SO_2_. The final fraction of the sulfated component is 17% in the high-Cu sample, and 22% in the low Cu/CHA sample. After the exposure to SO_2_, we find around 50% of the Cu present as the linear [Cu^I^(NH_3_)_2_]^+^ complex, and 25% as fw-Cu^I^, indicating that 75% of the Cu in the catalysts has undergone reduction to Cu^I^. We note that the [Cu_2_^II^(NH_3_)_4_O_2_]^2+^-complex is no longer present after the exposure to SO_2_, consistent with the high reactivity of this complex with SO_2_. These results indicate that the reaction of the [Cu_2_^II^(NH_3_)_4_O_2_]^2+^-complex with SO_2_ is not significantly affected by the variation of Cu content.

### Exposure to SO_2_ in the presence of O_2_

Following the exposure of [Cu_2_^II^(NH_3_)_4_O_2_]^2+^ dimers to SO_2_, approximately half of the Cu species undergoes a transformation into [Cu^I^(NH_3_)_2_]^+^. It is well known that the exposure of [Cu^I^(NH_3_)_2_]^+^ to O_2_ leads to the formation of [Cu_2_^II^(NH_3_)_4_O_2_]^2+^ complexes,^11,13,14^ which, in turn, can react with SO_2_. Consequently, in order to increase the SO_2_ uptake, the samples were exposed to a mixture of SO_2_ + O_2_, as it makes possible the regeneration of [Cu_2_^II^(NH_3_)_4_O_2_]^2+^ complexes from [Cu^I^(NH_3_)_2_]^+^.

**Fig. 4:**
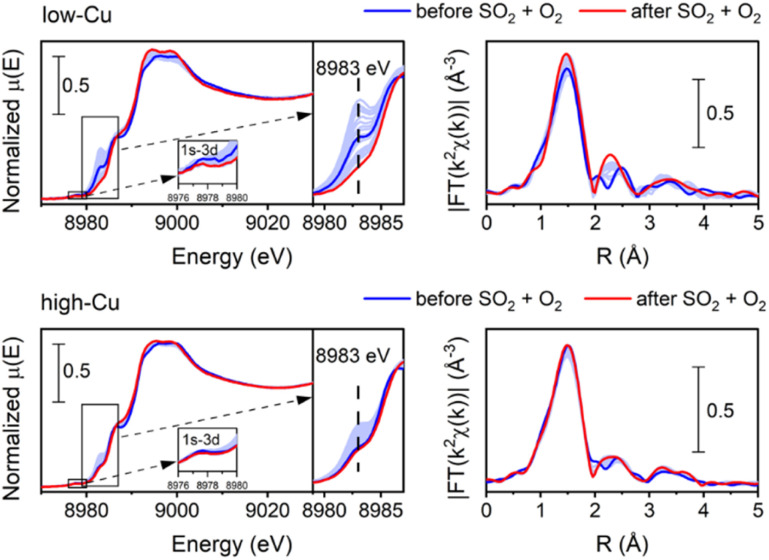
Cu K-edge XANES (a) and FT-EXAFS spectra (b) collected *in situ* during the exposure of the [Cu_2_^II^(NH_3_)_4_O_2_]^2+^ species to 360 ppm SO_2_ and 10% O_2_ at 200 °C for low-Cu (0.8 wt% Cu/CHA) and high-Cu (3.2 wt% Cu/CHA) catalysts.

Fig. 4 shows the evolution of XANES and EXAFS FT when exposing the [Cu_2_^II^(NH_3_)_4_O_2_]^2+^ complex to a mixture of 360 ppm SO_2_ and 10% O_2_ at 200 °C. XANES spectra reveal a slight initial increase and subsequent decrease of the peak at 8983 eV. This indicates that transient Cu^I^ species are formed in the process, which react further with the oxygen to form Cu^II^, resulting in the final oxidation state of Cu being +2. This is further corroborated by the 1s–3d transition at 8978 eV indicating the presence of Cu^II^, as this transition is not present in Cu^I^.

The intensity of the first peak of the EXAFS FT for the final spectrum after exposure to SO_2_ + O_2_ is close to the initial intensity, indicating that the average coordination number of the first shell of Cu remains close to four. That shows that 4-coordinated Cu species are dominant after the exposure to SO_2_ + O_2_. Moreover, the second peak becomes more pronounced, evidencing the presence of a relatively heavy neighbor at a well-defined distance in the second shell of Cu. The possible candidates are Si or Al from the zeolitic framework or S in case of formation of sulfated species.

**Fig. 5:**
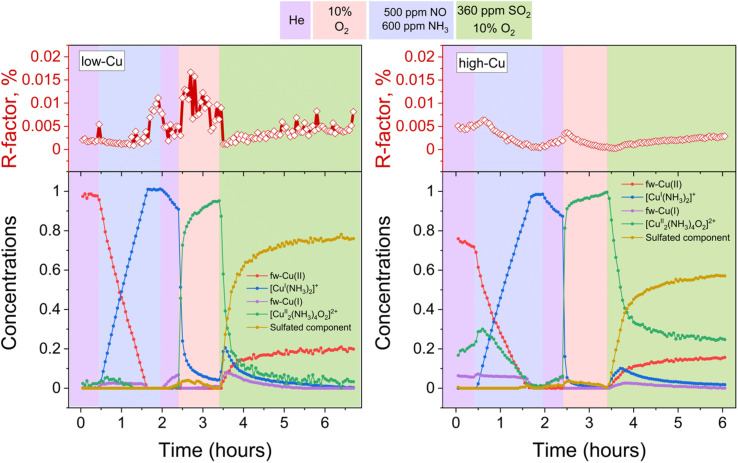
Quantification of Cu compounds during the *in situ* Cu K-edge XANES measurements from LCF, using the reference spectra shown in Fig. 2. Upper panels: *R*-factors of the linear combination fits. Lower panels: concentration profiles for the different Cu species during reduction in NH_3_ + NO, the formation of the [Cu_2_^II^(NH_3_)_4_O_2_]^2+^-complex, and exposure of the [Cu_2_^II^(NH_3_)_4_O_2_]^2+^-complex to SO_2_ + O_2_, for the low-Cu (left) and high-Cu (right) catalysts.

Fig. 5 displays the results of the combined MCR-ALS and LCF fitting of the XANES spectra collected during the formation of the [Cu_2_^II^(NH_3_)_4_O_2_]^2+^ followed by the exposure to the SO_2_ + O_2_ mixture. Compared to the results obtained after exposure to SO_2_ in the absence of O_2_ using the same set of reference spectral components, the fraction of the sulfated component has become significantly larger, especially for the low-Cu sample. Furthermore, both samples show a transient increase of fw-Cu^I^ and [Cu^I^(NH_3_)_2_]^+^ components in the beginning of the exposure to SO_2_ + O_2_, but very rapidly their concentration goes to zero. Interestingly, for the high-Cu sample, the significant fraction of component assigned to the [Cu_2_^II^(NH_3_)_4_O_2_]^2+^ complex remains even after the exposure to SO_2_ + O_2_ (see the Discussion). Finally, for both the low-Cu and the high-Cu catalysts, the fw-Cu^II^ component appears at the final stage of the process.

### Alternating exposure to SO_2_ and O_2_

**Fig. 6:**
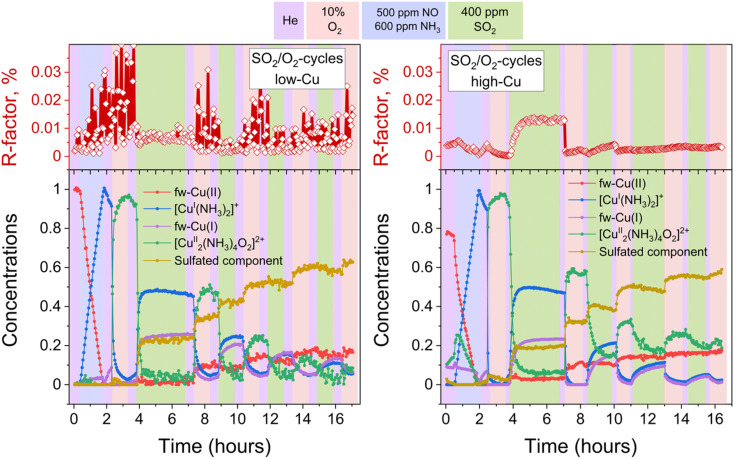
Quantification of Cu compounds during the *in situ* Cu K-edge XANES measurements from linear combination fits, using the reference spectra shown in Fig. 2. Upper panels: *R*-factors of the linear combination fits. Lower panels: concentration profiles for the different Cu species during reduction in NH_3_ + NO, the formation of the [Cu_2_^II^(NH_3_)_4_O_2_]^2+^ complex, and consequent exposures of the [Cu_2_^II^(NH_3_)_4_O_2_]^2+^ complex to SO_2_ and O_2_, for the low-Cu (left) and high-Cu (right) catalysts.

To discern the individual effects of SO_2_ and O_2_, a third experiment was conducted for both the low-Cu and high-Cu samples, wherein the dimers were exposed to alternating switches between SO_2_ and O_2_; the results of the LCF are shown in Fig. 6. Upon exposure to SO_2_ in the first two cycles, the [Cu_2_^II^(NH_3_)_4_O_2_]^2+^ complexes underwent decomposition, resulting in the formation of fw-Cu^I^, [Cu^I^(NH_3_)_2_]^+^ and the species corresponding to the sulfated component. However, in the subsequent 3rd and 4th cycles, the sulfated component did not exhibit significant growth during the exposure to SO_2_.

In the case of the low-Cu sample, the conversion of [Cu_2_^II^(NH_3_)_4_O_2_]^2+^ complexes at each step was nearly complete. In contrast, for the high-Cu sample, almost complete conversion was observed only after the initial exposure to SO_2_. Conversely, during each consequent exposure to SO_2_, a significant amount of the component previously assigned to the [Cu_2_^II^(NH_3_)_4_O_2_]^2+^ complex remained. This “unreactive” [Cu_2_^II^(NH_3_)_4_O_2_]^2+^ component forms after the first exposure of the sulfated sample to oxygen and, after subsequent exposure to SO_2_, stabilizes at the same level as after exposure of the high-Cu catalyst to SO_2_ + O_2_ (Fig. 5). At each subsequent exposure to SO_2_, less and less Cu^I^ is formed, concomitantly with a decreasing amount of the “reactive” [Cu_2_^II^(NH_3_)_4_O_2_]^2+^ component.

### Uptake of SO_2_ monitored by XAQ and SO_2_-TPD

The presence of oxygen during the SO_2_ exposure not only affects the final oxidation state of the Cu, but also leads to an increased uptake of SO_2_. This increased amount of SO_2_ in the catalyst can be quantified by X-ray adsorbate quantification (XAQ)^23^ and SO_2_-TPD.

**Fig. 7:**
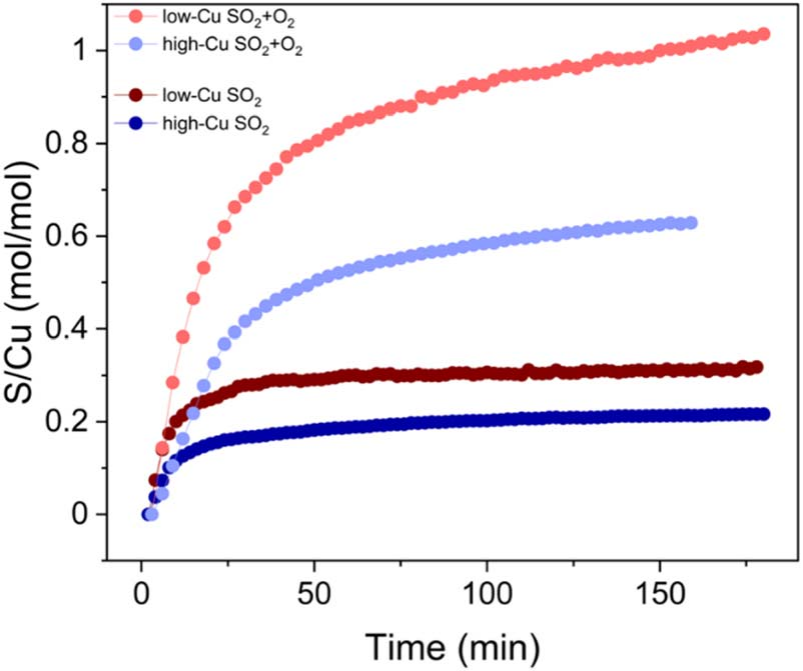
S/Cu ratio calculated from XAQ during exposure to SO_2_ and SO_2_ + O_2_ for low-Cu (0.8 wt% Cu/CHA) and high-Cu (3.2 wt% Cu/CHA) catalysts.

The XAQ technique relies on the phenomenon that the total absorption of X-rays depends on the composition of the sample. Therefore, the increase in total X-ray absorption during SO_2_ exposure reflects the increase in the amount of SO_2_ in the sample. This quantitative information was used to calculate the S/Cu ratios *in situ* during the exposure of the samples to the gas mixtures containing SO_2_. Fig. 7 shows the measured XAQ signals for the low Cu and high Cu catalysts during exposure to SO_2_ and SO_2_ + O_2_. All S/Cu curves have similar shape, exhibiting rather fast changes in the first 30–40 minutes of the measurement, followed by a much slower growth at a later stage. The final S/Cu levels reached in the presented experiments are 0.32 for the low-Cu sample and 0.22 for the high-Cu sample. In the presence of O_2_, the final levels increase to S/Cu = 1.04 and 0.63 for the low-Cu and high-Cu catalysts, respectively.

**Fig. 8:**
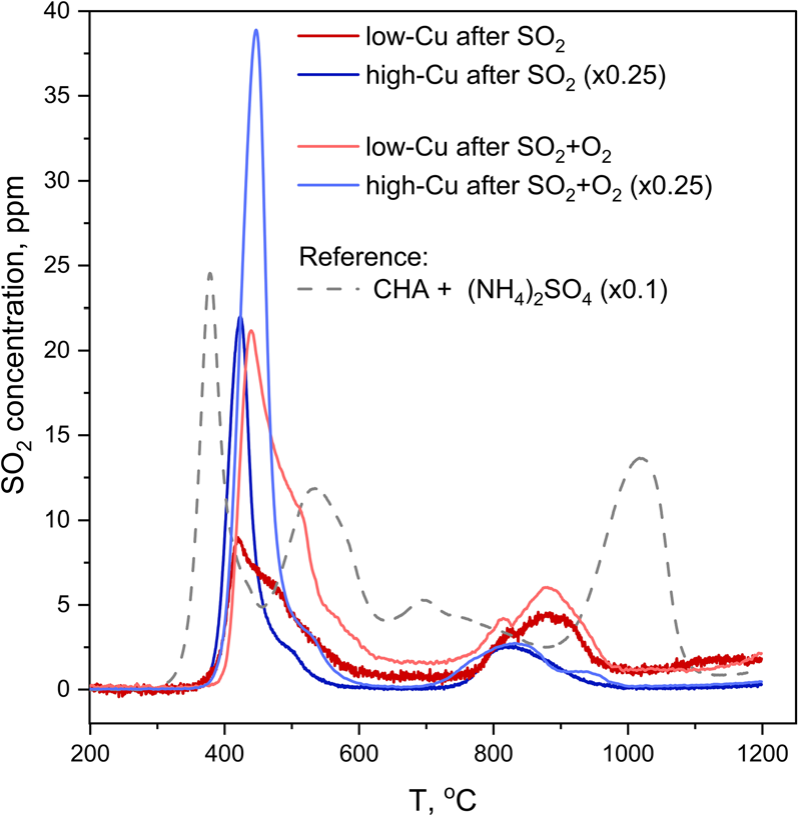
SO_2_-TPD profiles collected after exposure of the catalysts to SO_2_ (red and blue lines) and SO_2_ + O_2_ (orange and light blue lines) in comparison to a reference SO_2_-TPD curve of a Cu-free CHA zeolite impregnated with 20 wt% (NH_4_)_2_SO_4_, downscaled ×10. Pre-treatment is the same as for the procedures followed by *in situ* XAS. The curves for the high Cu catalysts (3.2 wt% Cu/CHA) (blue and light blue lines) are downscaled ×4.

The second method that was employed to measure SO_2_ uptake is SO_2_-TPD, where the desorption of SO_2_ is recorded as a function of temperature during heating of the catalyst. In contrast to XAQ, where the amount of SO_2_ adsorbed on the catalyst is monitored *in situ*, SO_2_-TPD measures the amount of SO_2_ released from a saturated sample. Fig. 8 shows the SO_2_-TPD data for the two catalysts after exposure to SO_2_ and SO_2_ + O_2_; the SO_2_-TPD of a Cu-free CHA impregnated with (NH_4_)_2_SO_4_ is included for comparison. All desorption curves for the Cu-CHA catalysts show a desorption feature in the range 400–600 °C, and one in the range 750–1000 °C. Because these profiles are clearly different from that of the adsorbed (NH_4_)_2_SO_4_ on the Cu-free zeolite reference, we conclude that the SO_2_ in the Cu-CHA catalysts predominantly interacts with the Cu, without a significant amount of free (NH_4_)_2_SO_4_. This is in good agreement with the conclusion that SO_2_ mainly reacts with the [Cu_2_^II^(NH_3_)_4_O_2_]^2+^ complex. The features in the range 750–1000 °C remain largely unaffected by the presence of O_2_, while the peak around 420 °C becomes larger, and shows a slight shift towards higher temperatures. Overall, SO_2_-TPD results indicate that the presence of O_2_ leads to a larger amount of SO_2_ in the Cu-CHA catalysts, and that SO_2_ binds predominantly to the Cu ions in the zeolite, in agreement with our earlier results.^16^

**Fig. 9:**
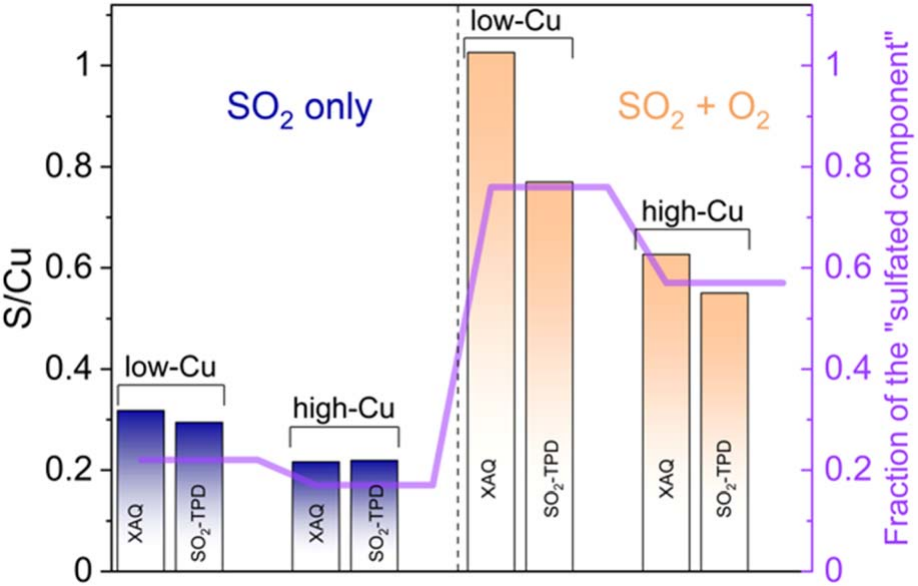
S/Cu ratios in the low-Cu (0.8 wt% Cu/CHA) and high-Cu (3.2 wt% Cu/CHA) samples after exposure to SO_2_ and SO_2_ + O_2_ obtained from XAQ and SO_2_-TPD compared to the concentration of the sulfated component obtained from the XANES LCF.

XAQ and TPD show that exposure to SO_2_ + O_2_ leads to a greater sulfur uptake compared to the exposure to only SO_2_ (Fig. 9). Importantly, the S/Cu molar ratio is in good correspondence with the concentration of the sulfated component in the XANES LCF, which confirms the assignment of the latter mainly to sulfated species.

### Sulfur K-edge XANES and Kα XES

**Fig. 10:**
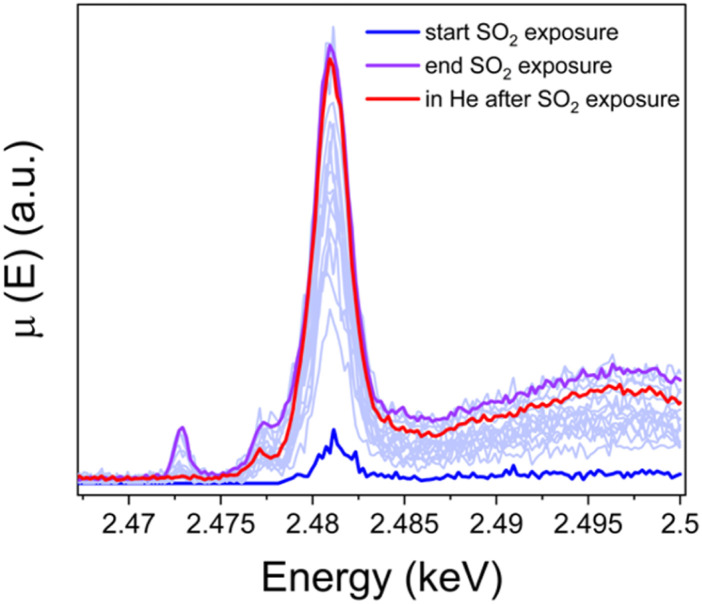
S K-edge XANES spectra of the high-Cu catalyst (3.2 wt% Cu/CHA) collected *in situ* during exposure of the [Cu_2_^II^(NH_3_)_4_O_2_]^2+^ complex to SO_2_ at 200 °C and in He after exposure to SO_2_.

**Fig. 11:**
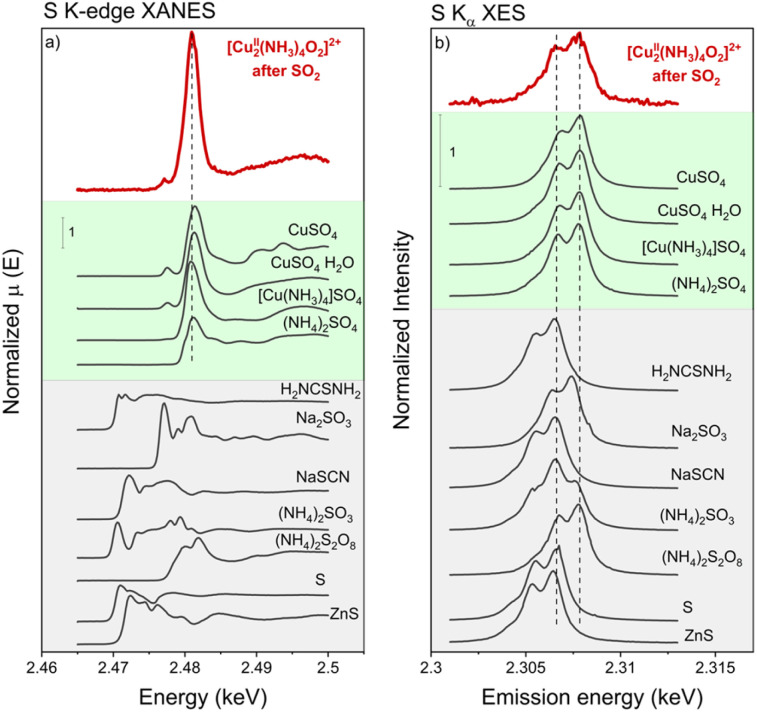
S K-edge XANES spectra of the [Cu_2_^II^(NH_3_)_4_O_2_]^2+^ complex exposed to SO_2_ and of the reference compounds. K-alpha XES spectra of the [Cu_2_^II^(NH_3_)_4_O_2_]^2+^ complex exposed to SO_2_ and references. High-Cu catalyst (3.2 wt% Cu/CHA).

To resolve the oxidation state of sulfur and the configuration of sulfur species in the sulfated Cu-CHA catalyst we measured S K-edge XANES during the exposure of the [Cu_2_^II^(NH_3_)_4_O_2_]^2+^ complex to SO_2_ and Kα-XES spectra at the end of the exposure. Fig. 10 shows the evolution of S K-edge XANES spectra during exposure of the [Cu_2_^II^(NH_3_)_4_O_2_]^2+^ complex to SO_2_. The spectra were collected in HERFD mode. The increase of the edge jump corresponds to the increasing concentration of sulfur, which means that S is accumulated in the sample. From the position of the edge and the shape of the spectrum we can identify the oxidation state of S and possible local environment by comparing with references. Fig. 11a shows that the sulfur in the sample predominately exists in the S^6+^ oxidation state, forming an SO_4_^2−^ group. Fig. 11b presents Kα XES spectra of the [Cu_2_^II^(NH_3_)_4_O_2_]^2+^ complex after exposure to SO_2_ in comparison with reference compounds. The positions of the two features at 2.3066 and 2.3078 keV in the Kα XES of the complex agree well with the references containing S^6+^ in the SO_4_^2−^ group, which is in line with the findings from S K-edge XANES.

### The sulfated component

From our measurements, we can identify the [Cu^I^(NH_3_)_2_]^+^, fw-Cu^II^, fw-Cu^I^ and reactive [Cu_2_^II^(NH_3_)_4_O_2_]^2+^ complexes, even though the exact structure of the framework-bound complexes is still under debate. Following the exposure of the Cu-CHA catalysts to SO_2_, both in the presence and in the absence of O_2_, a new species, whose spectrum we designated a “sulfated component”, appears.

**Fig. 12:**
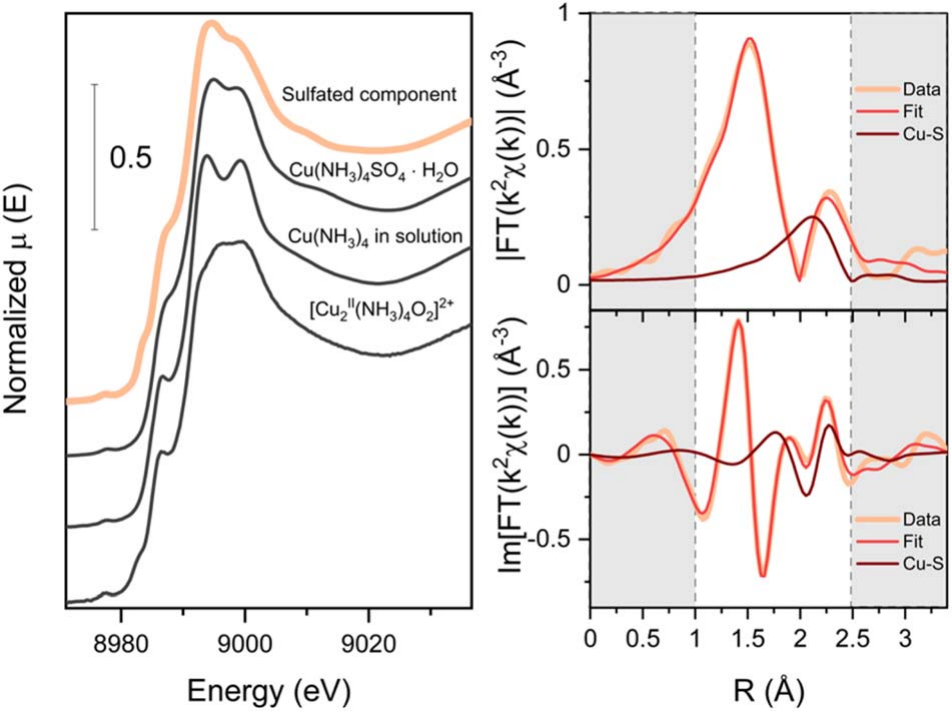
Comparison of the sulfated component obtained with MCR-ALS with the Cu K-edge XANES spectra of the [Cu(NH_3_)_4_]SO_4_·H_2_O, [Cu_2_^II^(NH_3_)_4_O_2_]^2+^ complex inside the CHA and Cu^II^_2_(NH_3_)_4_ in solution (left); the fitting results for the reconstructed EXAFS of the sulfated component (right).

In order to elucidate the structure of the species associated with the sulfated component, we compared the spectrum generated by MCR-ALS with the experimental spectra of three references: [Cu^II^(NH_3_)_4_]SO_4_·H_2_O, the [Cu_2_^II^(NH_3_)_4_O_2_]^2+^ complex formed in Cu-CHA and [Cu_2_^II^(NH_3_)_4_]^2+^ complex in solution (Fig. 12). The sulfated component has striking similarity to the spectrum of Cu(NH_3_)_4_SO_4_·H_2_O. Therefore, we propose that the species that give rise to the sulfated component contain 4-coordinated Cu^II^ in the square-planar environment similar to the one of Cu(NH_3_)_4_SO_4_, where a square-planar Cu^II^ ion is coordinated to four NH_3_ ligands. It is also possible that a geometrically similar Cu^II^ configuration with mixed NH_3_/O ligands is realized in the zeolite, since the XANES spectrum is expected to be very similar. Because the S atoms are not directly coordinated to the Cu in the structure of the [Cu^II^(NH_3_)_4_]SO_4_, and the Cu–S distance is about 4.6 Å, it is not possible to determine the precise location of the S atom in the sulfated species in the zeolite based on XANES data alone.

To locate the S atoms in the sulfated species, we extracted the EXAFS part of the sulfated component by subtracting the weighted reference spectrum for fw-Cu^II^ from the spectrum after exposure of the low-Cu catalyst to SO_2_ and O_2_ (see Fig. 4 and 5), using the weight coefficients derived from the LCF. We then fitted the EXAFS spectrum of the sulfated component with two N and two O in the first shell and S in the second shell. The fitting results are reported in Table 1. The resulting fit in comparison with the EXAFS of the sulfated component with a highlighted contribution of the Cu–S path is presented in Fig. 12. From this analysis, we find a Cu–S distance of 2.58 Å, which is much shorter than in the Cu(NH_3_)_4_SO_4_·H_2_O reference compound (4.65 Å). This means that the structure of the sulfated species is different from Cu(NH_3_)_4_SO_4_·H_2_O.

**Table 1:** Results of the EXAFS fitting for sulfated component

Path	*N*	*S*_0_^2^	*σ* ^2^, Å^2^	*R*, Å	*E*_0_, eV	*R*-Factor
Cu–O	2	0.9(1)	0.002(2)	1.94(2)	2.4	0.02
Cu–N	2		0.003(3)	2.09(2)		
Cu–S	1		0.006(2)	2.58(2)		

## Discussion

### The unreactive part of the [Cu_2_^II^(NH_3_)_4_O_2_]^2+^ component

For the high-Cu catalyst, the concentration of the component assigned to [Cu_2_^II^(NH_3_)_4_O_2_]^2+^ complexes does not go to zero during repeated exposures to SO_2_ followed by exposure to O_2_, remaining above 18% after each exposure to SO_2_ (see Fig. 6).

The first explanation for such behavior may be that a fraction of the [Cu_2_^II^(NH_3_)_4_O_2_]^2+^ complexes become inaccessible for SO_2_ and therefore do not react. This interpretation resembles the mechanism for deactivation of Cu-CHA catalysts as proposed by Bjerregaard *et al.*^17^ A further consequence of this interpretation is that it limits the uptake of SO_2_ in a Cu-CHA catalyst with a sufficiently high Cu content (*ca.* 3 wt%), while, at the same time, a part of the Cu does not react with SO_2_. Such a conclusion is in good agreement with earlier measurements of a limited uptake of SO_2_ in Cu-CHA catalysts,^3,4^ and the observation that Cu-CHA catalysts show a residual activity after saturation with SO_2_.^3^ With such an interpretation, the structure of the [Cu_2_^II^(NH_3_)_4_O_2_]^2+^ complex remains unchanged, which is directly reflected in the XANES spectra.

The second explanation for the persistence of the component assigned to the [Cu_2_^II^(NH_3_)_4_O_2_]^2+^ complex involves a transformation of the [Cu_2_^II^(NH_3_)_4_O_2_]^2+^ complex to a different structure, having similar coordination of the Cu-ions. A possibility is the formation of peroxo–dicopper complexes Cu_*x*_O_*y*_ attached to the framework,^28^ which can be formed by exposure of the Cu-CHA to O_2_ at 400–500 °C. Such species are expected to have a very similar XANES spectrum to the [Cu_2_^II^(NH_3_)_4_O_2_]^2+^ complexes, because coordination of the Cu-ions is similar, and oxidation state is the same. Therefore, XANES spectra of such framework-bound complexes may be difficult to distinguish from those of the [Cu_2_^II^(NH_3_)_4_O_2_]^2+^ complex by MCR-ALS analysis, which merges them into a single component. Such an interpretation is supported by the observation that the component assigned to [Cu_2_^II^(NH_3_)_4_O_2_]^2+^ complexes appears also during the initial oxidation of the high-Cu catalyst at 500 °C (green curve in Fig. 6, right panel, at *t* = 0) with the concentration of around 20%, which is very close to the concentration observed after exposure to SO_2_ + O_2_. Clearly, it is not possible to form [Cu_2_^II^(NH_3_)_4_O_2_]^2+^ complexes at the activation stage, since the required NH_3_ is not yet available and the temperature is too high. As such moieties are essentially fw-Cu^II^ species, we expect that their reactivity towards SO_2_ is very low, as we have shown in our previous work,^16^ thus resulting in an accumulation of these species in the sample.

### Unraveling the mechanism of the sulfation reaction

The results presented above have some implications for a mechanism describing the reaction of SO_2_ with Cu-CHA catalysts. Sulfur XES and XANES show that S is stored in the sample as S^6+^ within (SO_4_)^2−^ tetrahedra. This indicates an oxidation of SO_2_ by the [Cu_2_^II^(NH_3_)_4_O_2_]^2+^ complex. A formation of an (SO_4_)^2−^ group in the absence of O_2_ in the gas implies oxygen transfer from the [Cu_2_^II^(NH_3_)_4_O_2_]^2+^ complexes to the SO_2_ molecule.

In the reaction of SO_2_ with the [Cu_2_^II^(NH_3_)_4_O_2_]^2+^ complexes in the absence of O_2_, the S/Cu ratio reaches only around 25% (Fig. 9). Nevertheless, all Cu in the catalyst changes its coordination environment, since all [Cu_2_^II^(NH_3_)_4_O_2_]^2+^ complexes are converted (Fig. 3). The fact that all Cu in the catalyst is affected at a S/Cu uptake of 0.25 suggests that a single SO_2_ molecule reacts with at least two [Cu_2_^II^(NH_3_)_4_O_2_]^2+^ complexes. This requires either sufficient mobility of the [Cu_2_^II^(NH_3_)_4_O_2_]^2+^ complexes or that first a mobile intermediate product is formed in the reaction between SO_2_ and the first complex, which then reacts with a second [Cu_2_^II^(NH_3_)_4_O_2_]^2+^. Since the mobility of the bulky [Cu_2_^II^(NH_3_)_4_O_2_]^2+^ complex is expected to be limited, the formation of a smaller mobile intermediate product seems more likely.

Bringing these considerations together, we arrive at a reaction pathway consisting of two steps. In the first step (eqn (1)), SO_2_ reacts with a [Cu_2_^II^(NH_3_)_4_O_2_]^2+^ complex, the peroxo bond breaks, a mobile SO_a_X intermediate forms, and Cu^II^ reduces to Cu^I^. The Cu^I^ appears in the form of [Cu^I^(NH_3_)_2_]^+^ and fw-Cu^I^, as indicated by the LCF (Fig. 3). A possible candidate for the mobile intermediate SO_a_X is SO_3_, formed by oxidation of SO_2_, upon the formation of Cu^I^-species. It is known that SO_3_ has a similar effect on the NH_3_-SCR activity of Cu-CHA as SO_2_,^3,20,29,30^ which would be consistent with the proposed reaction scheme. However, our data do not allow the exact structure of the mobile SO_a_X complex to be determined, so the formation of SO_3_ remains to be proven (or ruled out) experimentally.

1 Cu_2_^II^(NH_3_)_4_O_2_ + SO_2_ → Cu^I^(NH_3_)_2_ + fw-Cu^I^ + SO_a_X + …

2 SO_a_X + Cu_2_^II^(NH_3_)_4_O_2_ → Cu^I^(NH_3_)_2_ + Cu^II^SO_4_Z + …

In the second step (eqn (2)), the mobile SO_a_X intermediate reacts with another [Cu_2_^II^(NH_3_)_4_O_2_]^2+^ complex, breaking the peroxo-bond to form the (SO_4_)^2−^ group within the sulfated Cu-species associated with sulfated XAS component (Cu^II^SO_4_Z) and another linear diamino complex [Cu^I^(NH_3_)_2_]^+^. Z in the sulfated species Cu^II^SO_4_Z may comprise O, framework O or NH_3_ to result in a square-planar coordination of Cu with 4-ligands proven by our EXAFS and XANES results (Fig. 12). This two-step reaction scheme is in line with the Cu concentration profiles obtained by the LCF analysis in the case of exposure to SO_2_ in the absence of O_2_. Note that eqn (1) and (2) aim to summarize the experimental findings in this article, and therefore do not represent a complete mechanism of the reaction of SO_2_ with the Cu-CHA catalyst.

When the sample is exposed to SO_2_ in the presence of O_2_, we observe an initial transient formation of the same Cu^I^ intermediates, as in case of the exposure to SO_2_ alone (Fig. 5). This indicates that O_2_ reoxidizes the [Cu^I^(NH_3_)_2_]^+^ complexes into new [Cu_2_^II^(NH_3_)_4_O_2_]^2+^ species. In this way, the Cu^I^-species formed in the reaction with SO_2_ become available again for further reaction with SO_2_, which explains the increased SO_2_ uptake compared to exposure to SO_2_ without oxygen. This is corroborated by the results obtained with alternating exposure of the Cu-CHA catalysts to SO_2_ and O_2_ (Fig. 6), showing alternating conversion of the [Cu_2_^II^(NH_3_)_4_O_2_]^2+^ complexes in the SO_2_ phases and their reconstruction from the Cu^I^ during the exposures to O_2_.

**Fig. 13:**
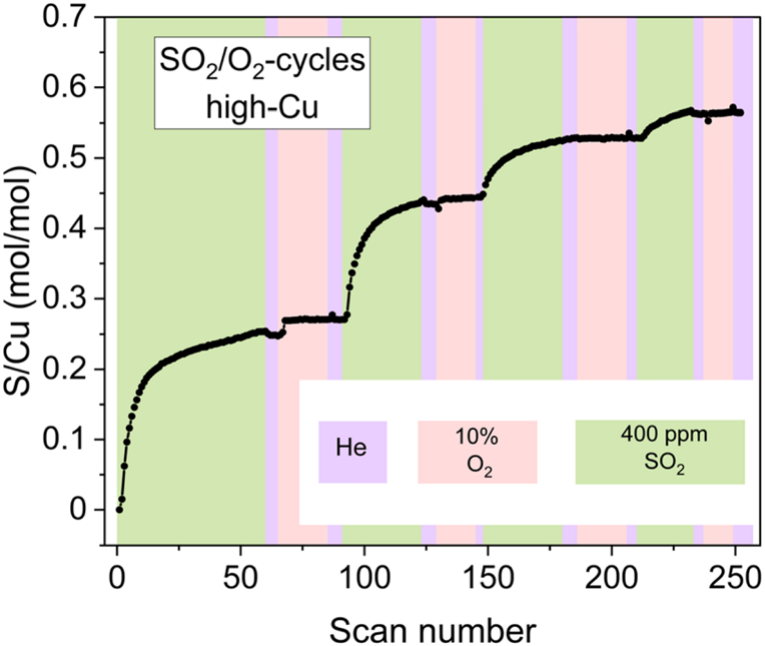
The evolution of the S/Cu ratio during the SO_2_/O_2_-cycles for the high-Cu sample (3.2 wt% Cu/CHA) deduced from the XAQ signal.

However, the exposure of the sample to alternating SO_2_/O_2_ cycles (Fig. 6) also reveals that the growth of the sulfated component occurs not only in SO_2_ phases but also when the sample is exposed to O_2_. Moreover, during the later stages of the cycles (3rd and 4th cycles), the growth of the sulfated component is observed only in the presence of O_2_, while the exposure to SO_2_ at this stage does not result in the formation of the sulfated component at all. Nonetheless, even at this stage the reactive [Cu_2_^II^(NH_3_)_4_O_2_]^2+^ complexes do undergo decomposition in the presence of SO_2_, resulting in sulfur uptake, as evidenced by the evolution of the S/Cu ratio obtained from the *in situ* XAQ signal (Fig. 13).

A possible explanation for this effect is that at the late stages of the cycles, when the reactive Cu species are few, it is less likely to have two [Cu_2_^II^(NH_3_)_4_O_2_]^2+^ complexes close enough to perform the second step of the sulfation reaction (eqn (2)). Therefore, the mobile sulfur species SO_a_X formed in the first step (eqn (1)) can be converted into the sulfated Cu species only after the stock of [Cu_2_^II^(NH_3_)_4_O_2_]^2+^ complexes is replenished upon the exposure to O_2_. It is possible that in such regime SO_a_X undergo further transformations (*e.g.* reacting with NH_3_ or NH_4_^+^) before reacting with newly formed [Cu_2_^II^(NH_3_)_4_O_2_]^2+^ and yielding the sulfated Cu species, but the obtained data do not allow unambiguous identification of the corresponding reaction pathways. Nonetheless, the observed effect serves as indirect confirmation of the multi-step nature of the sulfation process involving at least two [Cu_2_^II^(NH_3_)_4_O_2_]^2+^ complexes per SO_2_.

## Conclusions

In this study, we applied *in situ* XAS at Cu and S K-edges, S Kα XES, XAQ and SO_2_-TPD to investigate the interaction mechanism between the [Cu_2_^II^(NH_3_)_4_O_2_]^2+^ complex in the Cu-CHA catalyst and SO_2_.

Upon reacting the [Cu_2_^II^(NH_3_)_4_O_2_]^2+^ complex with SO_2_, a mixture of fw-Cu^I^ (approximately 1/4 of total Cu), [Cu^I^(NH_3_)_2_]^+^ complexes (approximately 1/2) and a new sulfated Cu^II^ compound (approximately 1/4) are formed. The presence of oxygen in the gas mixture with SO_2_ enhances the reaction, leading to higher concentrations of the sulfated species and an increased S/Cu ratio in the sample. This effect is explained by reoxidation of the [Cu^I^(NH_3_)_2_]^+^ species to the reactive [Cu_2_^II^(NH_3_)_4_O_2_]^2+^ complexes. The catalysts with 0.8 wt% Cu/CHA and 3.2 wt% Cu/CHA demonstrated similar results.

Following a multi-technique experimental approach, the structure of the Cu and S local environment of sulfated species accumulated in the sample was uncovered. Copper in the sulfated species exists as Cu^2+^ and adopts a square-planar coordination with four light ligands in the first coordination shell, which, most probably, are NH_3_ and O. Sulfur in the sulfated species is in the S^6+^ oxidation state, forming an SO_4_ group. The sulfur atom is located in the second shell of Cu at an approximate distance of 2.6 Å, suggesting that Cu and S are connected through two oxygen ligands.

## Data availability

The datasets presented in this article are available at the ESRF repository: https://cloud.esrf.fr/s/2HQDGDp3w7DX4zX.

## Author contributions

The manuscript was written through contributions of all authors.

## Conflicts of interest

There are no conflicts to declare.

## Supplementary Material

SC-014-D3SC03924B-s001
