# Supplementary material for: Elucidating the reaction mechanism of SO2 with Cu-CHA catalysts for NH3-SCR by X-ray absorption spectroscopy
Source: Chem Sci. 2023 Oct 10;14(41):11521–31. doi: 10.1039/d3sc03924b (PMC10599480; doi:10.1039/d3sc03924b)
Supplement: SC-014-D3SC03924B-s001 [file SC-014-D3SC03924B-s001.pdf]

Electronic Supplementary Material (ESI) for Chemical Science.
This journal is © The Royal Society of Chemistry 2023

# Elucidating the reaction mechanism of $SO_2$ with Cu-CHA catalysts for $NH_3$-SCR by X-ray absorption spectroscopy

# Supporting information

Anastasia Yu. Molokova[1,2], Reza K. Abasabadi[2,3], Elisa Borfecchia[2], Olivier Mathon[1], Silvia Bordiga[2], Fei Wen[4], Gloria Berlier[2], Ton V.W. Janssens[3*] and Kirill A. Lomachenko[1*]

[1] *European Synchrotron Radiation Facility, 71 avenue des Martyrs, CS 40220, 38043 Grenoble Cedex 9, France*

[2] *Department of Chemistry and NIS Centre, University of Turin, via Giuria 7,10125 Turin, Italy*

[3] *Umicore Denmark ApS, Kogle Allé 1, 2970 Hørsholm, Denmark*

[4] *Umicore AG & Co, Rodenbacher Chaussee 4, 63457 Hanau, Germany*

## Table of Contents

1. Experimental details ........ 1
   1.1. In situ XAS ........ 1
   1.2. S K-edge XANES and S Kα XES ........ 2
   1.3. XAQ ........ 2
   1.4. $SO_2$-TPD ........ 2
   1.5. Experimental procedures ........ 2
2. Low Cu catalyst: XANES and EXAFS results ........ 4
3. Linear combination fitting (LCF) and Multivariate curve resolution (MCR) ........ 5
   3.1. MCR and reference spectra for LCF ........ 5
   3.2. Fitting results ........ 7
4. References ........ 7

## 1. Experimental details

### 1.1. In situ XAS

The XAS experiments were performed at BM23 beamline of the ESRF. The storage ring was operating in 4-bunch top-up mode at 20 to 30 mA maximum current. Measurements were performed in transmission mode at Cu K-edge, using a double-crystal Si(111) monochromator

moving in a continuous mode. Acquisition time for one XAS spectrum was 3 min, energy range of 8800-10000 eV, energy step of 0.3 eV. A pair of flat Si mirrors at 2.8 mrad angle was used for harmonic rejection. Ionization chambers were used to detect incident and transmitted photons. $I_0$ chamber was filled with 1.57 bar $N_2$, $I_1$ and $I_2$ with 0.33 bar Ar, all three of them topped-up with He to the total pressure of 2 bar(a). The spectra of the Cu reference foil were measured together with the sample and then used for alignment. The XAS data were analysed using Athena and Artemis programs from Demeter package[1] and Larch[2] code.

## 1.2. S K-edge XANES and S Kα XES

The HERFD XANES and XES experiments at S K-edge were performed at ID26 beamline of the ESRF. The storage ring was operating in uniform top-up mode at 200 mA maximum current. The measurements were done using the tender x-ray emission spectrometer (TEXS) with five Si(111) crystal analysers, placed in a vacuum chamber to minimize X-ray absorption along the sample-analysers-detector path. Acquisition time for one HERFD XANES spectrum was 5 s, energy range of 2.465-2.51 keV, emission energy corresponding to the maximum of Kα emission line. For the XES Kα measurements incident radiation energy was fixed at 2.65 keV. XES Kα data were collected integrating 1 s per point, each point collected at the fresh spot of the sample.

## 1.3. XAQ

The X-ray adsorbate quantification (XAQ) data were obtained by monitoring the change of the total absorption coefficient before Cu K edge (at 8885 eV, averaging the region of 8820-8950 eV) during the interaction $SO_2$. The change of the absorption coefficient was attributed to the adsorption of $SO_2$. The corresponding number of moles of $SO_2$ per unit area of the pellet was calculated by XAFS mass code[3] and, knowing the parameters of the sample pellet (mass, surface area and Cu content), converted into Cu/S ratio.

## 1.4. $SO_2$-TPD

The $SO_2$-TPD was performed with the samples of the same material sulfated following the same procedures as employed in the XAS experiment. The $SO_2$-TPD was carried out with simultaneous thermal analyser (STA 449 F3 Jupiter, NETZSCH), which is coupled with an infrared (FTIR) spectrometer (MKS 2030, MKS Instruments) for evolved gas analysis. The sample was first kept in nitrogen gas flow 100 ml/min at 50°C for 30 mins and then heated up to 1200°C with 10 K/min. The sample mass is typically 25 mg.

## 1.5. Experimental procedures

The goal of each experimental procedure was to expose the $[Cu^{II}_2(NH_3)_4O_2]^{2+}$ complex to $SO_2$, a mixture to $SO_2/O_2$/He and cycles of $SO_2$ and $O_2$. The detailed schemes containing the information on the temperatures, gas compositions and timing are presented in Figures S1-S3.

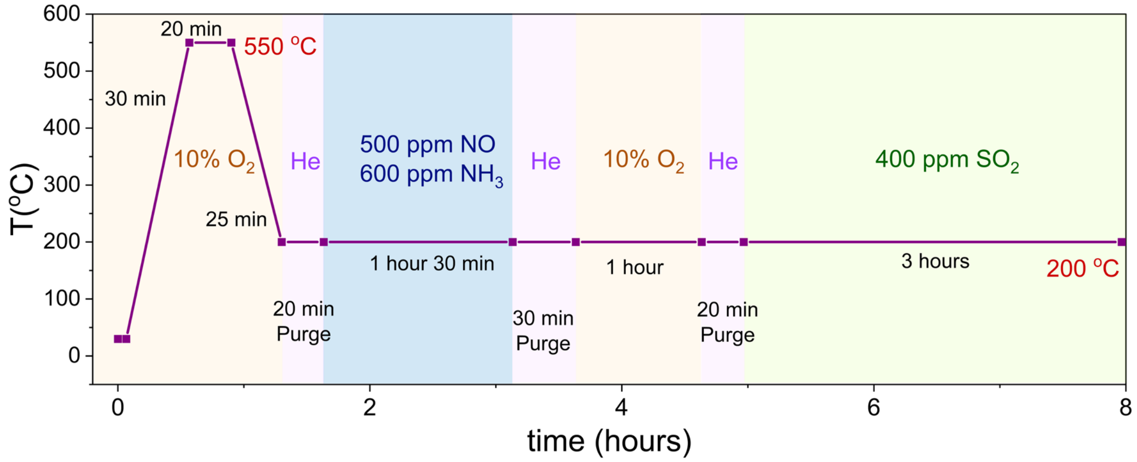


Figure S1. Scheme of the experimental protocol of the exposure of the $[Cu^{II}_2(NH_3)_4O_2]^{2+}$ to $SO_2$.

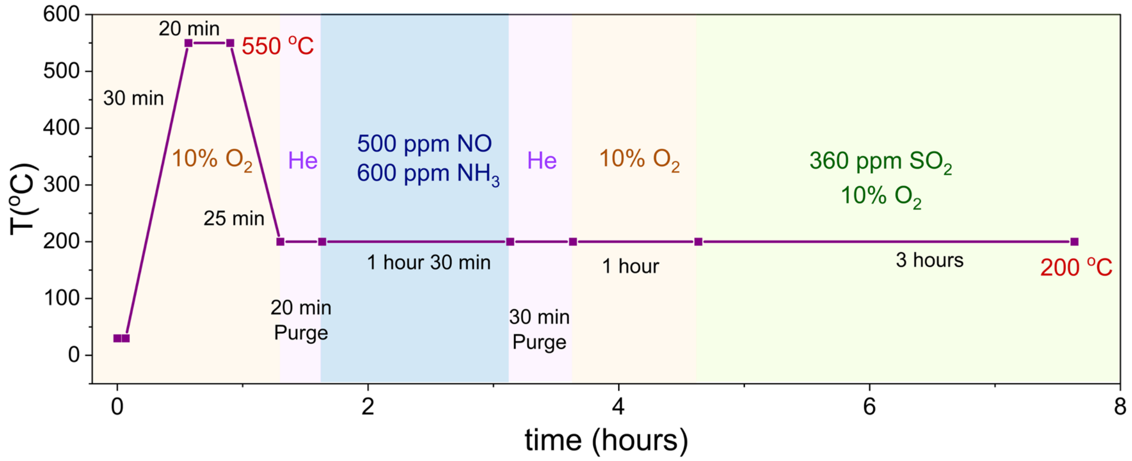


Figure S2. Scheme of the experimental protocol of the exposure of the $[Cu^{II}_2(NH_3)_4O_2]^{2+}$ to a mixture of $SO_2/O_2/He$.

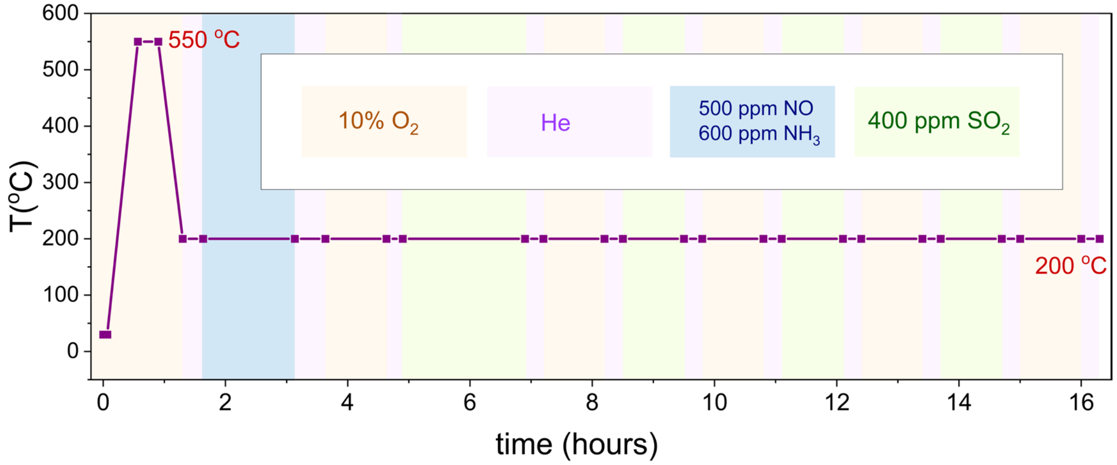


Figure S3. Scheme of the experimental protocol of the exposure of the $[Cu^{II}_2(NH_3)_4O_2]^{2+}$ to the cycles of $SO_2$ and $O_2$.

## 2. Low Cu catalyst: XANES and EXAFS results

Table S1. Pre-treatment procedures and resulting Cu species.

| Procedure | Conditions | Dominant Cu state | Designation in the text and figures | Reference |
|---|---|---|---|---|
| 1 | 1% $H_2$ at 400 °C; cooling to 200 °C in He | fw-$Cu^I$ | fw-$Cu^I$ | 4 |
| 2 | 500 ppm NO + 600 ppm $NH_3$ at 200 °C | mobile $[Cu^I(NH_3)_2]^+$ | $[Cu^I(NH_3)_2]^+$ | 4 |
| 3 | 500 ppm NO + 600 ppm $NH_3$ at 200 °C; heating to 550 °C in He; cooling back to 200 °C in He | fw-$Cu^I$ (after thermal treatment of $[Cu^I(NH_3)_2]^+$) | $[Cu^I(NH_3)_2]^+$ + T | 4 |
| 4 | 10% $O_2$ at 200 °C | fw-$Cu^{II}$ | fw-$Cu^{II}$ | 5 |
| 5 | 500 ppm NO + 600 ppm $NH_3$ at 200 °C; He purge; 10% $O_2$ at 200 °C | mobile $[Cu^{II}_2(NH_3)_4O_2]^{2+}$ dimer | $[Cu^{II}_2(NH_3)_4O_2]^{2+}$ | 6 |
| 6 | 600 ppm $NH_3$ at 200 °C | mixed* | $Cu^{II}$ + $NH_3$ | 7 |

For the low-Cu catalyst (0.8 wt% Cu/CHA) we observe similar results as presented before for the high-Cu catalyst (3.2 wt% Cu/CHA) [7].

Figure S4 shows the evolution of Cu K-edge XANES and EXAFS spectra during the exposure of the pre-treated Cu-CHA catalyst to 400 ppm $SO_2$/He flow at 200 °C. When Cu is in a $Cu^I$ state, the spectra exhibit only minor changes during the exposure to $SO_2$, indicating that the $SO_2$ does not readily interact with the $Cu^I$ ions (Procedures 1-3). Therefore, these Cu species are less likely to react with $SO_2$ alone during the catalytic cycle. The situation remains similar if the sample is only exposed to $O_2$ (Procedure 4), indicating the low reactivity of the fw-$Cu^{II}$ species towards $SO_2$. However, the result is clearly different for $Cu^{II}$ in the presence of $NH_3$. Cu species obtained after interaction with $NH_3$ (Procedures 5 and 6) show significant rearrangement in the local structure and changes in the Cu oxidation state upon interaction with $SO_2$. In both cases the exposure to $SO_2$ causes a pronounced increase of the XANES peak at 8983 eV, characteristic for linear $Cu^I$ complexes,[8-10] and a drop of the first shell intensity in the EXAFS FT.

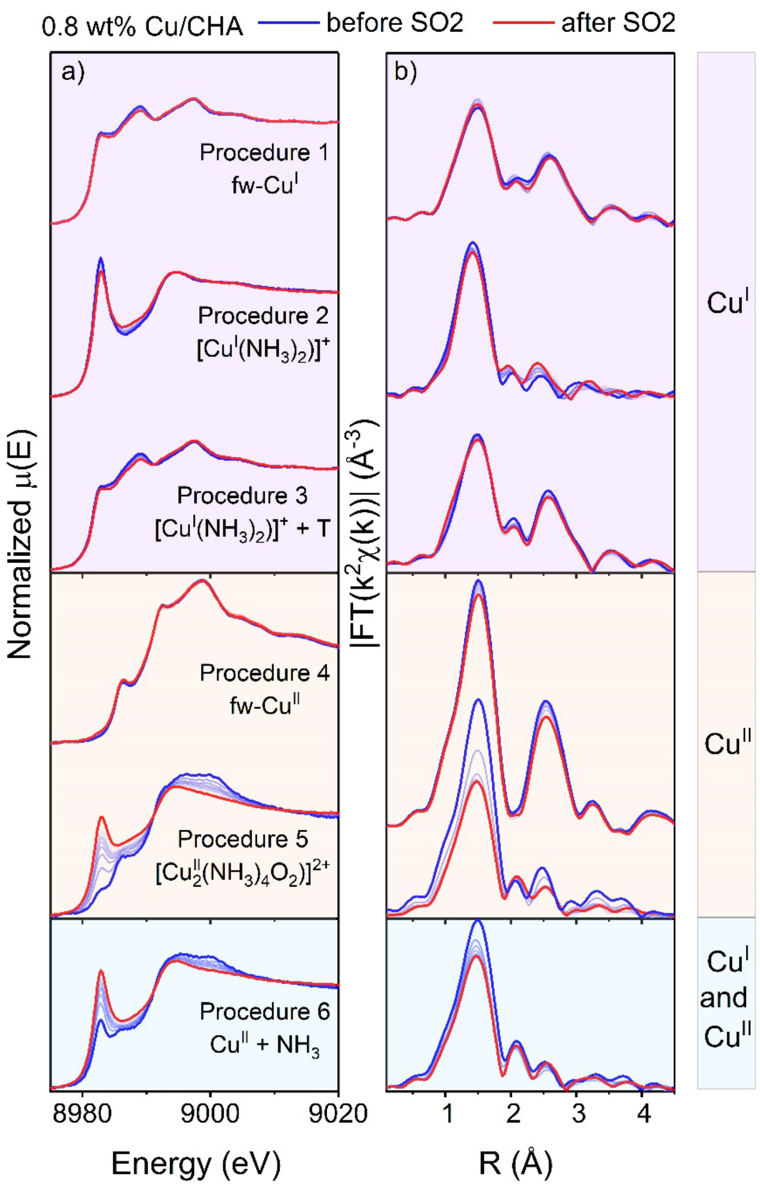


Figure S4. Cu K-edge XANES (a) and FT-EXAFS spectra (b) collected *in situ* during the exposure of Cu species obtained in Procedures 1-6 to $SO_2$ at 200 °C. Low Cu catalyst (0.8 wt% Cu/CHA)

# 3. Linear combination fitting (LCF) and Multivariate curve resolution (MCR)

## 3.1. MCR and reference spectra for LCF

To determine the spectral composition resulting from the reaction of the $[Cu_2^{II}(NH_3)_4O_2]^{2+}$ complex with $SO_2$ and $SO_2$+$O_2$ we applied a combination of MCR and LCF.

The MCR analysis utilized a dataset consisting of six sets of spectra: two sets for the procedure involving $SO_2$ only, two sets for the procedure involving $SO_2$+$O_2$, and two sets for $SO_2$/$O_2$-cycles. The two sets for each procedure included data for low-Cu and high-Cu catalysts. By resolving these six datasets together, we were able to obtain the known spectra of fw-$Cu^{II}$, $[Cu^{I}(NH_3)_2]^+$, fw-$Cu^{I}$ and $[Cu_2^{II}(NH_3)_4O_2]^{2+}$ and a new spectrum which we assigned as "sulfated component". Some spectra generated by MCR have artefacts, so we took experimental spectra as references instead. The choice of references is explained in details below.

We chose one set of references for all datasets for LCF. The first reference is fw-$Cu^{II}$. The spectrum obtained with MCR has some artefacts in the pre-edge region, so we took an experimental spectrum (Figure S5a). We chose the spectrum of the low-Cu sample, because it was closer in shape to the MCR generated spectrum, and therefore can be considered as a purer reference in this case.

The experimental spectra of $[Cu^{I}(NH_3)_2]^+$ were very similar to the spectrum generated by MCR, so we took the experimental spectrum of $[Cu^{I}(NH_3)_2]^+$ for the sample with higher Cu content due to lower level of noise.

The spectra of $[Cu_2^{II}(NH_3)_4O_2]^{2+}$ complex of low-Cu and high-Cu samples were very similar, so we employed the spectrum of the high-Cu catalyst as a reference, because of its lower noise.

The spectrum corresponding to fw-$Cu^{I}$ was taken from the MCR, because the spectra of the two samples collected in the conditions that favored the formation of fw-$Cu^{I}$ species were noticeably different (Figure S5b). However, since the spectral features were qualitatively the same, the difference in the experimental spectra is most probably due to the different content of minority species (such as fw-$Cu^{II}$), whereas the dominant component deduced by MCR and corresponding to pure fw-$Cu^{I}$ species is likely to be the same for both samples.

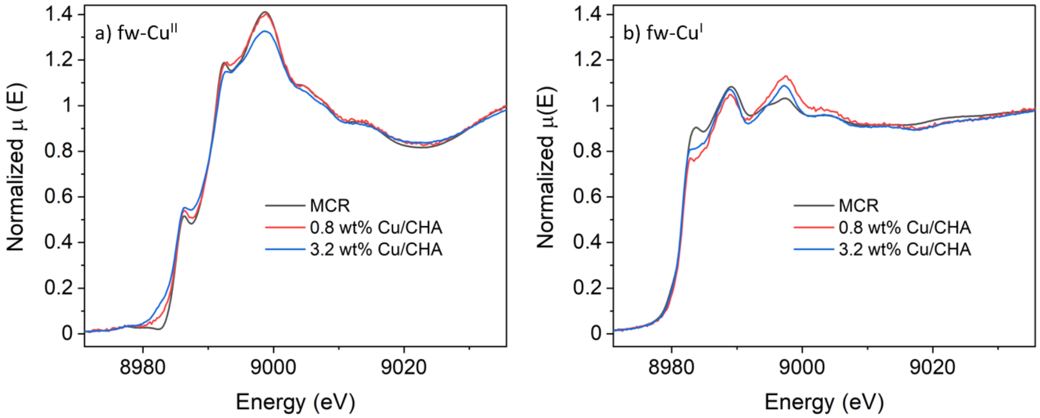


Figure S5. Comparison of the spectra of fw-$Cu^{II}$ (a) and fw-$Cu^{I}$ (b) generated by MCR and experimental spectra of the low-Cu and high-Cu catalysts, for choosing the reference components for LCF.

## 3.2. Fitting results

The fit results of the final spectra after exposure to $SO_2$ and $SO_2$+$O_2$ presented in Figure S6 show good agreement between the spectra and fits for all procedures.

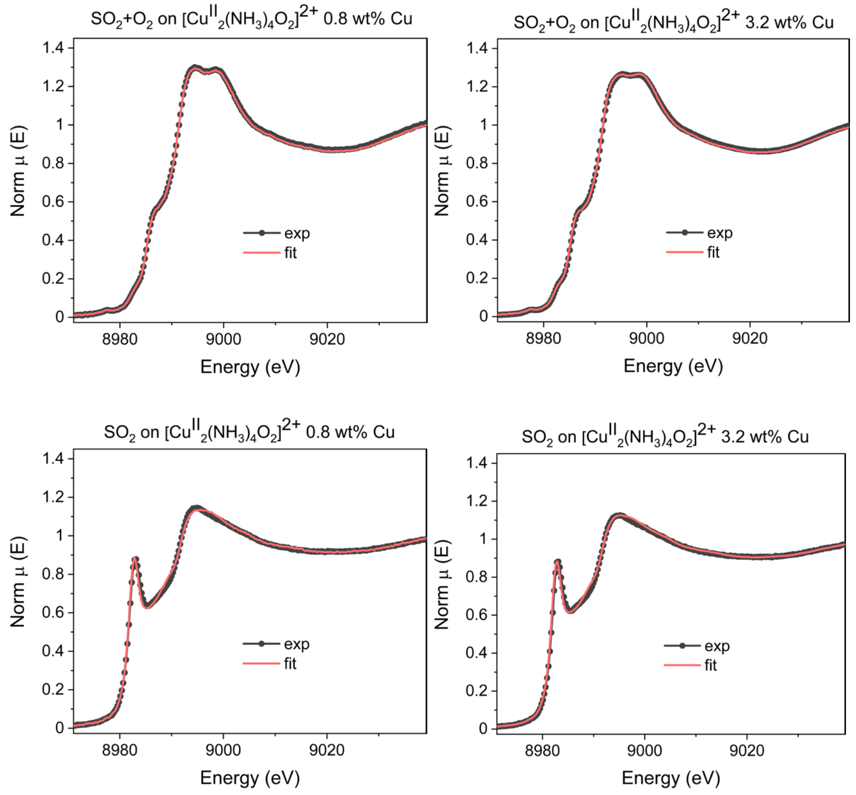


Figure S6. Resulting linear combination fits of the XANES spectra after the interaction with $SO_2$ and $SO_2$+$O_2$.

# 4. References


1. B. Ravel and M. Newville, ATHENA and ARTEMIS Interactive Graphical Data Analysisusing IFEFFIT, *Physica Scripta*, 2005, **T115**, 1007–1010.
2. M. Newville, Larch: An Analysis Package For XAFS And Related Spectroscopies. *Journal of Physics: Conference Series*, 2013, **430**, 012007.
3. K. Klementiev and R. Chernikov, XAFSmass: a program for calculating the optimal mass of XAFS samples, *Journal of Physics: Conference Series*, 2016, **712**, 012008.
4. E. Borfecchia, C. Negri, K. A. Lomachenko, C. Lamberti, T. V. W. Janssens and G. Berlier, Temperature-dependent dynamics of NH3-derived Cu species in the Cu-CHA SCR catalyst, *React Chem Eng*, 2019, **4**, 1067-1080.
5. A. Martini, E. Borfecchia, K. A. Lomachenko, I. A. Pankin, C. Negri, G. Berlier, P. Beato, H. Falsig, S. Bordiga and C. Lamberti, Composition-driven Cu-speciation and reducibility in Cu-CHA zeolite catalysts: a multivariate XAS/FTIR approach to complexity, *Chem Sci*, 2017, **8**, 6836-6851.

6. C. Negri, T. Selleri, E. Borfecchia, A. Martini, K. A. Lomachenko, T. V. W. Janssens, M. Cutini, S. Bordiga and G. Berlier, Structure and Reactivity of Oxygen-Bridged Diamino Dicopper(II) Complexes in Cu-Ion-Exchanged Chabazite Catalyst for NH3-Mediated Selective Catalytic Reduction, *J. Am. Chem. Soc.*, 2020, **142**, 15884-15896.
7. A. Y. Molokova, E. Borfecchia, A. Martini, I. A. Pankin, C. Atzori, O. Mathon, S. Bordiga, F. Wen, P. N. R. Vennestrøm, G. Berlier, T. V. W. Janssens and K. A. Lomachenko, SO2 Poisoning of Cu-CHA deNOx Catalyst: The Most Vulnerable Cu Species Identified by X-ray Absorption Spectroscopy, *JACS Au*, 2022, **2**, 787–792.
8. L. S. Kau, D. J. Spira-Solomon, J. E. Penner-Hahn, K. O. Hodgson and E. I. Solomon, X-ray absorption edge determination of the oxidation state and coordination number of copper. Application to the type 3 site in Rhus vernicifera laccase and its reaction with oxygen, *J. Am. Chem. Soc.*, 1987, **109**, 6433-6442.
9. T. V. W. Janssens, H. Falsig, L. F. Lundegaard, P. N. R. Vennestrom, S. B. Rasmussen, P. G. Moses, F. Giordanino, E. Borfecchia, K. A. Lomachenko, C. Lamberti, S. Bordiga, A. Godiksen, S. Mossin and P. Beato, A Consistent Reaction Scheme for the Selective Catalytic Reduction of Nitrogen Oxides with Ammonia, *ACS Catal.*, 2015, **5**, 2832-2845.
10. F. Giordanino, E. Borfecchia, K. A. Lomachenko, A. Lazzarini, G. Agostini, E. Gallo, A. V. Soldatov, P. Beato, S. Bordiga and C. Lamberti, Interaction of NH3 with Cu-SSZ-13 Catalyst: A Complementary FTIR, XANES, and XES Study, *J. Phys. Chem. Lett.*, 2014, **5**, 1552-1559.
